# Inactivated Poliovirus Vaccine Booster Reduces the Likelihood of COVID-19 Outcomes in Individuals Primed with Oral Poliovirus Vaccination

**DOI:** 10.3390/vaccines12030219

**Published:** 2024-02-20

**Authors:** Brittany A. Comunale, Robin J. Larson, Yea-Jen Hsu, Erin Jackson-Ward, Chisom Azodoh, Aditi Singh, Lilly D. Engineer

**Affiliations:** 1 Department of Health Policy and Management, Johns Hopkins Bloomberg School of Public Health, Johns Hopkins University, Baltimore, MD 21205, USA; 2 Geisel School of Medicine at Dartmouth, Hanover, NH 03755, USA; 3 Department of Palliative Medicine, Dartmouth-Hitchcock Medical Center, Lebanon, NH 03756, USA; 4 Cedars-Sinai Medical Center, Los Angeles, CA 90048, USA; 5 Department of Biological Sciences, University of California, San Diego, CA 92161, USA; 6 Department of Anesthesiology and Critical Care Medicine, Johns Hopkins School of Medicine, Baltimore, MD 21205, USA

**Keywords:** SARS-CoV-2 infection, COVID-19, poliovirus, mucosal immunity, drug repositioning, vaccines, immunology, OPV

## Abstract

**Introduction:** Prior research explores whether seasonal and childhood vaccines mitigate the risk of SARS-CoV-2 infection. Although there are trials investigating COVID-19 infection in response to the effects of the oral poliovirus vaccine (OPV), there has been no prior research assessing COVID-19 outcomes in recently immunized adults with the inactivated poliovirus vaccine (IPV). **Methods:** SARS-CoV-2 infection and COVID-19 symptoms were analyzed across a cohort of 282 adults who received an IPV booster. Bivariate and multivariate regression models explored associations among variables related to vaccination histories and COVID-19 outcomes. **Results:** One year post-IPV inoculation, participants who had never received OPV were more likely to test positive for SARS-CoV-2 and experience COVID-19 symptoms, compared to those who had previously received OPV (OR = 3.92, 95%CI 2.22–7.03, *p* < 0.001; OR = 4.45, 95%CI 2.48–8.17, *p* < 0.001, respectively). Those who had never received OPV experienced COVID-19 symptoms for 6.17 days longer than participants who had previously received OPV (95%CI 3.68–8.67, *p* < 0.001). Multivariate regression modeling indicated COVID-19 vaccination did not impact SARS-CoV-2 infection or COVID-19 symptoms in this sample of adults who had recently received IPV. **Discussion:** Findings suggest IPV may boost mucosal immunity among OPV-primed individuals, and COVID-19 vaccination may not provide additional protection among those who had received IPV. Future, larger-scale studies should measure the extent of protective effects against COVID-19 to inform public health policies in resource-deficient settings.

## 1. Introduction

Even though 3 years have passed since the onset of the severe acute respiratory syndrome coronavirus 2 (SARS-CoV-2) pandemic, uncertainties about vaccination efficacy, protective effects against novel variant strains, and the duration of induced antibodies still linger. Despite the questions, the pathogenesis of SARS-CoV-2, the virus that causes coronavirus disease 2019 (COVID-19), is well understood, and may be used to help inform risk mitigation and vaccination strategies. SARS-CoV-2 most commonly spreads when viral particles are transmitted through respiratory droplets from one human to another. As a first step to transmission, the S1 subunit of the SARS-CoV-2 spike protein attaches to receptors of enzymes in the upper respiratory tract, called angiotensin-converting enzymes 2 (ACE2) [1]. Then, the S2 spike protein subunit prompts viral fusion and assists the virus in entering host cells in the epithelial cell lining. This process consequently enables the RNA-dependent RNA polymerase (RdRp) protein to replicate viral RNA strands, promoting disease progression and inflammation [2,3]. This process of viral replication can occur in various parts of the body that, in turn, promote associated symptoms. For instance, when the process occurs in alveoli in the lungs, COVID-19 disease may manifest in the form of a cough [4]. Since ACE2 is expressed not only in the lungs but also throughout the body, such as the gastrointestinal tract, it is unsurprising that COVID-19 symptoms are often related to gastric distress (i.e., nausea, vomiting, diarrhea) [5,6]. The fact that the manifestations of COVID-19 may be seen through various pathways suggests a need for diverse immunity.

The human body has two main lines of defense from infection: innate and adaptive (also known as acquired or specific) immunity. Innate immunity is provided by the body’s composition, ranging from physical protections (i.e., skin or mucous membranes), to chemical barriers (i.e., acidic environments to combat microorganisms), and cellular defenses (i.e., pre-existing proteins or enzymes that respond to invading nonspecific pathogens) [7,8]. Adaptive immunity, on the other hand, is not pre-existing and must be acquired through passive or active exposure [9]. Obtaining immunity passively means gaining antibodies either (1) as a fetus via placenta or an infant through breastfeeding or (2) by receiving immune serum with existing antibodies, meaning that a person does not have to produce antibodies on their own. Acquiring immunity actively, on the other hand, entails obtaining antibodies either through (1) natural infection or direct exposure to the virus or pathogen or (2) vaccination [10]. While innate immunity provides immediate, temporary protection from nonspecific pathogens, adaptive immunity provides specific and long-lasting immunity, ranging from a few years to a lifetime [11]. The degree of protection from vaccination may depend on the design and mechanism of the vaccine.

The only prophylactic measures currently available for SARS-CoV-2 are COVID-19 vaccines, and all vaccines target the same protein, the S-glycoprotein or “spike” protein, to generate an immune response against SARS-CoV-2 [12]. The main concern with targeting this surface protein, though, is antigenic drift, the process through which RNA viruses drift from their original form and evolve into variant strains, which stems from viral gene point mutations that modify surface protein structures [13]. Thus, older COVID-19 vaccines that were designed to target the original, Alpha strain’s spike protein have become less effective over time against the mutating variant that is causing infections [14]. Without access to the more recently developed bivalent (Alpha/Omicron BA.4/5) vaccine, resource-deficient countries must continue to utilize the original, less effective vaccine. By contrast, the United States has the significant advantage of being in a position to target a SARS-CoV-2 spike protein that closely resembles that of the most recently circulating variant strain. An updated COVID-19 vaccine from Pfizer-BioNTech and Moderna that targets the spike protein of Omicron variant XBB.1.5 was made available in the United States in Fall 2023 [15]. Since most regions of the world do not have the resources to constantly design and administer updated vaccines for emerging variants, there is a need to combat SARS-CoV-2 in an equitable, accessible, and efficient manner that may complement currently deployable resources.

Instead of focusing on a mutable protein located on the surface, therapeutic agents that target more ‘conserved,’ or non-surface, viral regions that are structurally preserved across variants may be more effective long-term. The RdRp protein, responsible for RNA synthesis and viral replication, is one such protein that is highly conserved across RNA viruses despite changes in genetic sequences [16]. Accordingly, drugs that were created with the intention of targeting the RdRp protein of one RNA virus are now being used to mitigate COVID-19 disease progression. For instance, remdesivir, an intravenous RdRp inhibitor developed for the Ebola virus, and molnupiravir, an oral drug designed for the influenza virus, are commonly used to treat symptomatic COVID-19 patients [17,18,19,20]. Since in silico (computer simulations), in vitro (laboratory experiments), and in vivo (studies performed on living organisms) evidence has demonstrated the efficacy of RdRp inhibitors against SARS-CoV-2 variants, it is unsurprising that these drugs have been successful across the various waves of SARS-CoV-2 strains [21,22,23,24,25,26,27,28,29,30,31,32]. In addition to the exploration of RdRp inhibitors as COVID-19 disease treatments, there has also been interest in using anti-RdRp agents in a prophylactic manner (i.e., to prevent disease and/or provide some protection before an exposure may occur).

When it comes to prophylaxis and COVID-19, researchers have explored the potential effect of common vaccinations against RNA viruses that replicate via RdRp proteins, such as influenza, on COVID-19 morbidity and mortality [33,34,35,36,37,38]. For instance, a matched case-control study of nearly 31,000 healthcare workers in Qatar found that influenza vaccination significantly reduced SARS-CoV-2 infection and the likelihood of experiencing severe COVID-19 disease [39]. Similarly, a retrospective cohort analysis of nearly 75,000 patients found influenza vaccination to be associated with lower risk of sepsis, stroke, deep vein thrombosis, and COVID-19 hospitalizations [40]. Such protective effects may be due to trained immunity, a process also known as innate immune memory, where innate immune cells demonstrate enhanced immunological response to vaccinations and re-infections due to epigenetic and metabolic reprogramming [41,42,43]. Moreover, influenza vaccines that are administered intranasally stimulate even more diverse immune responses than vaccines that are given intramuscularly, because mucosal and systemic immunity are induced, aiding the proliferation of memory CD8 T-cells [44].

The poliovirus vaccine is another common vaccine that targets RdRp viral replication; it is available in both inactivated intramuscular and live oral versions and has been associated with lower risks of SARS-CoV-2 infection [45]. The oral vaccine provides stronger local intestinal (mucosal) and nasopharyngeal immunity than IPV can offer [46]. For example, when wild poliovirus interacts with proteolytic enzymes from the stomach (i.e., trypsin), viral antigens are modified, and only OPV can develop secretory and humoral antibodies against these newly evolved antigens. This reaction occurs because the live vaccine enables an individual to be exposed to poliovirus antigens that infect mucosal surfaces and, thus, develop mucosal antibodies that provide local (intestinal) immunity at the site of infection, as well as memory cells that can respond to subsequent exposures. The immune response induced by IPV alone is not as diverse or robust [47,48]. Moreover, OPV has the ability to disrupt viral transmission between individuals and, therefore, is considered more effective at containing viral outbreaks in possible endemic regions, particularly resource-deficient countries [49].

Interestingly, countries that use OPV for their childhood vaccination campaigns have reported lower incidence rates of SARS-CoV-2 infection compared with those that solely use IPV [50]. Accordingly, nearly all studies examining the effects of poliovirus vaccination on COVID-19 morbidity and mortality have focused on OPV [51,52,53,54,55,56,57,58]. To date, the only published study examining the effects of IPV on SARS-CoV-2 RdRp is an in vitro retrospective analysis of polio-immune serum samples. Comunale et al., (2021) observed that SARS-CoV-2 RdRp antibodies are able to recognize one or more epitopes in poliovirus RdRp. Moreover, polio-immune sera may inhibit viral SARS-CoV-2 cytopathic activity [59]. Herein, a prospective clinical trial that inoculated 300 adults with IPV and documented participant-reported COVID-19 outcomes for 12 months is described in a secondary analysis. Factors related to vaccination histories are explored, including how influenza, IPV, OPV, and COVID-19 vaccinations may impact SARS-CoV-2 positive infection results and/or experiences related to COVID-19 symptoms 12 months post-IPV inoculation.

This study aims to further elucidate the factors related to vaccination histories that are associated with (1) a positive SARS-CoV-2 test result, (2) COVID-19 symptoms, and (3) the duration of COVID-19 symptoms among adults with no prior history of COVID-19 infection or vaccination. Such factors include, but are not limited to, whether or not participants received OPV as children, the number of COVID-19 vaccine shots received, and over what period of time.

Considering the findings of prior literature, the initial hypothesis of this study was that the incidence of SARS-CoV-2 infection, COVID-19 symptoms, and the duration of symptoms would be greater among those who had never received COVID-19, influenza, or OPV vaccinations and those whose most recent influenza or COVID-19 vaccinations were more than 6 months prior to SARS-CoV-2 exposures. Based on what was known about the impact of variants at the time of this study, it was expected that SARS-CoV-2 infection would be higher among those exposed to the Omicron variant strain compared to the Delta variant strain, and that the incidence of COVID-19 symptoms would be higher and for a longer duration among those exposed to the Delta variant strain compared to the Omicron variant strain.

## 2. Materials and Methods

### 2.1. Clinical Trial Design

The described study constitutes one component of a larger study under clinical trial NCT04639375, centering on a retrospective secondary analysis of self-reported COVID-19 outcome data by participants collected through phone interviews at 6 and 12 months post-enrollment [60].

**Figure 1:**
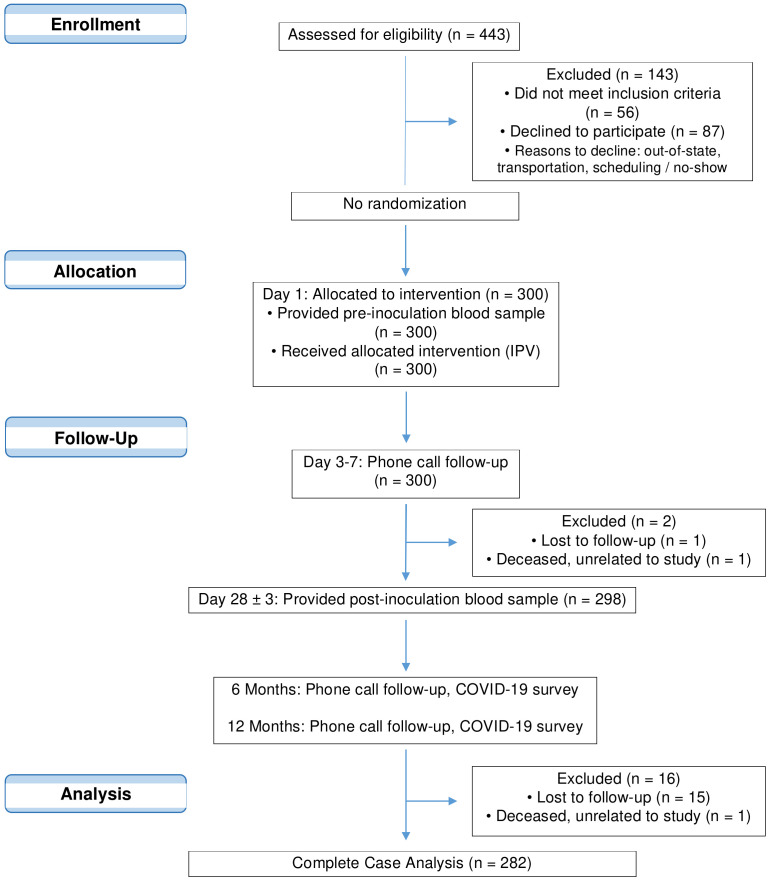
CONSORT flow diagram of clinical trial NCT04639375 and subsequent analyses.

The single-arm, open-label clinical trial pilot study administered IPV to 300 individuals located in Southern California from November 2020 to May 2021. Enrolled and eligible participants, aged 18 to 80, had no active infectious diseases, no prior COVID-19 vaccinations, and no IPV or OPV poliovirus vaccinations during the past 12 years. Once enrolled, deemed Day 1 of the trial, participants donated a baseline blood sample and received an IPV poliovirus injection (Figure 1). Participants were telephoned between Days 3 and 7 to report and record any adverse reactions due to the IPV injection. On Day 28 (+/− 3 days), participants returned to the clinic for a second blood draw. Participants were instructed to report any known exposures to SARS-CoV-2 or positive infection tests as soon as possible through phone or email. After Day 28, participants were permitted to receive COVID-19 vaccinations.

Clinical site staff contacted participants via phone 6 and 12 months after Day 1 to record any known COVID-19 exposures, COVID-19 related symptoms, and details surrounding the exposure, such as location and event. The trial concluded in May 2022. All information related to COVID-19 exposures and positive test results reported via email or phone during the prior 6 months were verified during the follow-up call. Participants were also asked about their vaccination histories, specifically if and when they received vaccinations for COVID-19, influenza, Tdap, and poliovirus. Participants that received COVID-19 vaccinations following the second clinic visit were asked to report the vaccine brand (Pfizer-BioNTech, Moderna, or Janssen [Johnson & Johnson]), vaccination dates, and number of vaccinations. In the case of prior polio vaccinations, the year(s) and type of vaccine were noted: OPV (sugar cube or liquid drop) or IPV (intramuscular injection). Clinical staff confirmed vaccination histories through the vaccination registry database, and, if accessible, participant-provided vaccination card copies.

Participants were contacted up to three times via phone and subsequently by certified letter for both 6- and 12-month check-ins, as per clinical trial protocol. The time, date, and success or failure for each attempted contact were recorded by the clinical trial coordinator. In cases where all contact attempts were ineffective, the participant was denoted as “lost to follow-up”.

### 2.2. Data Source

Clinical trial NCT04639375 was performed according to International Conference on Harmonisation Good Clinical Practice (ICH GCP) and the United States Code of Federal Regulations (CFR) applicable to clinical studies (45 CFR Part 46, 21 CFR Part 50, 21 CFR Part 56, 21 CFR Part 312, and/or 21 CFR Part 812). Approval from the institutional review board (Advarra) was secured for the study protocol, informed consent forms, recruitment materials, and all subject-facing materials. Written informed consent was provided by all participants. The trial was conducted in compliance with ICH GCP and the ethical standards described in the WMA Declaration of Helsinki.

Authors were not directly involved in data collection for clinical trial NCT04639375, but in the context of using these data as a subset of a larger study, the first and last authors obtained the necessary permissions from the clinical trial sponsor, EMO-Biotech, Inc., as well as the Johns Hopkins School of Public Health Institutional Review Board.

### 2.3. Ethics

The Johns Hopkins School of Public Health Institutional Review Board reviewed this secondary analysis study and deemed it exempt from the need for ethical review.

### 2.4. Measures and Variables

#### 2.4.1. Independent Variables

In total, one continuous and nine categorical variables were selected a priori for this study based on previous literature (Table 1). Participants were asked about their vaccination histories, as some vaccines, particularly COVID-19, influenza, and OPV, may decrease the risk of SARS-CoV-2 infection and severity of COVID-19 disease. Variables related to vaccination timing were included, because the duration of protection provided by these vaccinations can vary. Researchers have found that immunity derived from COVID-19 vaccinations wanes over time and can depend on which brand of COVID-19 vaccine was received, how many doses were administered, and which different SARS-CoV-2 variants the subject had been exposed to. Several multicenter studies have found that the Moderna vaccine not only is more protective against hospitalizations but also stimulates higher antibody levels compared to the Pfizer-BioNTech vaccine [61,62]. However, both are clinically effective in minimizing the danger of severe COVID-19 disease and enabling higher antibody levels than those induced by the Janssen (Johnson & Johnson) vaccine [63,64]. In terms of vaccine effectiveness across SARS-CoV-2 variants, studies have found lower efficacy of the primary series shots when patients were exposed to the Omicron variant compared with those exposed to the Delta variant [65,66,67,68]. Additionally, protection from COVID-19 vaccinations has also been noted to decrease after 3 or 4 months, and even more so after 6 months [61,66,67,68,69,70,71]. However, a combination of natural infection and COVID-19 vaccination may provide antibodies for up to 1 year [72]. In any event, researchers recommend additional COVID-19 vaccinations, to serve as “booster shots,” to maintain higher antibody levels [67,68].

In terms of immunity, it takes about 2 weeks to mount antibodies from influenza vaccines, with immunity peaking 2 to 4 weeks later and waning between 3 and 6 months after inoculation [73,74,75,76,77]. While it is not known how long poliovirus antibodies last, studies have shown that individuals can still be completely resistant to wild poliovirus 10 or even 18 years after receiving OPV, and some assert that protective effects could potentially last a lifetime [78,79,80]. As such, the variable Years Between Last Poliovirus Vaccination (IPV or OPV) Prior to Enrollment and SARS-CoV-2 Exposure was analyzed as a continuous factor. The variable Received Tetanus-Diphtheria-Pertussis (Tdap) Vaccine in the Last 5 Years was also included, because researchers have found that Tdap vaccinations can increase the sensitivity of memory T-cells and may reduce the severity of COVID-19 disease [81].

Clinical staff directly recorded all independent variables during 6- and 12-month phone check-ins, with the exception of two independent variables that were curated by the first author: (1) Number of COVID-19 Vaccines Received and (2) Exposure to COVID-19 Omicron or Delta Strains. The number of COVID-19 vaccines received for each participant was recorded based on source documentation collected by the clinical site staff, which described the brand of COVID-19 vaccine, the date of vaccination, and the series (i.e., two-dose primary, or booster). The four levels for this variable were created based on vaccination status: None (Not Vaccinated); One (Partially Vaccinated); Two (Fully Vaccinated); Three (Fully Vaccinated + Boosted). Even though participants who received the Janssen (Johnson & Johnson) vaccine (N = 10) technically only received a single injection, they were placed in the Two (Fully Vaccinated) category, having received the equivalent of two Moderna or Pfizer-BioNTech primary series shots. It is also important to note that only one booster COVID-19 vaccine was available to the public during the study timeframe, so the maximum number of COVID-19 vaccines a participant could have received was three.

Responses related to SARS-CoV-2 variant strains were ascertained by looking at a regional SARS-CoV-2 variant strain dashboard and denoting dominant strains by exposure dates [82].

#### 2.4.2. Dependent Variables

Three outcomes of interest were analyzed in this study: Participant Positive for SARS-CoV-2 (Yes/No); Experienced COVID-19 Symptoms (Yes/No); and COVID-19 Symptoms Duration (Days, continuous).

### 2.5. Missing Data

Eighteen participants (6.0%) did not respond to 6- and 12-month phone calls or certified letters and were classified as “lost to follow-up”. There were no discernable differences in the numbers or reasons for attrition. The median age and age range of those who discontinued the trial (51, 18–69 years) were comparable to participants who remained in the study (55, 18–80 years). An equal proportion of males and females (50.0%) were lost to follow-up. To ensure that adjustments in linear or logistic regression models resulted from new variables added to the model rather than a reduction in sample size due to missing values, data from these 18 participants were excluded to create a complete case analysis dataset. Considering that the attrition rate approached the widely accepted 5% threshold, the impact of missing data is likely negligible.

### 2.6. Statistical Analysis

RStudio (version 2022.12.0+353) was used to generate statistical models and analyses. Variables associated with vaccination histories were chosen before conducting analyses and were informed by prior literature investigating the severity of COVID-19, connections to RdRp activity, and/or an understanding of vaccine-derived immunity.

Logistic regressions were used for the two dichotomous variables of interest, Tested Positive for SARS-CoV-2 (Yes/No) and Experienced COVID-19 Symptoms (Yes/No); linear regression analyses were carried out for the continuous variable of interest, Duration of COVID-19 Symptoms.

First, bivariate regressions of each independent variable were performed to assess main effects on each dependent outcome of COVID-19. Interaction terms associated with the research questions were also examined for their main effects. Next, multivariate regressions using forward selection were performed through a phased approach to adjust for multiple influential factors and to address each research aim.

To assess if, when, and which vaccines may influence testing positive for SARS-CoV-2 infection (Aim 1), each independent variable was included in the various iterations of regression modeling using a phased approach. The first model was a bivariate logistic regression of Exposed to Omicron or Delta SARS-CoV-2 Strain and Tested Positive for SARS-CoV-2, because some researchers have noted that the Omicron variant strain is more easily transmissible than the Delta strain [83,84], ultimately increasing the chance of testing positive for SARS-CoV-2 infection. The second model was a bivariate regression of Received COVID-19 Vaccine (Yes/No) and Tested Positive for SARS-CoV-2, because the Centers for Disease Control and Prevention asserted in 2021 that COVID-19 vaccination could reduce the risk of SARS-CoV-2 infection by 91% [85]. Subsequent models added or subtracted each of the other independent variables to create new combinations of adjusted factors. Differences in odds ratios, standard error, and *p* values were investigated at each step to assess whether additional variables were introducing confounding factors to the primary relationship.

Furthermore, the Akaike information criterion (AIC) and Bayesian information criterion (BIC) were computed at each step to further assess the model fit. Models with smaller AIC and BIC values indicated potential parsimony while accurately fitting the data. Among these, the model with the lowest value was selected as the best fit. Likelihood ratio and Wald tests were also employed to determine whether regression coefficients for predictor variables and interaction terms in the final model significantly enhanced the overall fit, comparing models with and without the variable of interest. Odds ratios and 95% confidence intervals were reported for the final model, with *p* values less than 0.05 considered statistically significant.

The same methodological process was repeated to evaluate if, when, and which vaccines may influence whether or not participants experienced COVID-19 symptoms (Aim 2). The first logistic regression model was a bivariate regression of Exposed to Omicron or Delta SARS-CoV-2 Strain and Experienced COVID-19 Symptoms, since studies have suggested an increased likelihood of symptomatic infection upon exposure to the Delta variant, compared with more asymptomatic cases from the Omicron variant [86]. The second logistic regression model was a bivariate comparison of the Number of COVID-19 Vaccines Received and Experienced COVID-19 Symptoms, due to research assertions that the effectiveness of COVID-19 vaccines in reducing symptomatic SARS-CoV-2 infection may be dependent on how many doses one receives [66,67,68]. Once more, different combinations and iterations of the remaining independent variables were incorporated into subsequent models.

Finally, a comparable phased approach was employed to investigate the hypothesis that the duration of symptoms, indicating potential severity, may be affected by participants’ vaccination histories (Aim 3). The first linear regression model was a comparison of Exposed to Omicron or Delta Strain and Duration of COVID-19 Symptoms, because an analysis of a large healthcare system in Southern California, the same geographic region where the clinical trial analyzed herein was located, found that patients exposed to the Omicron variant strain had a lower risk of disease progression and severity than those exposed to the Delta variant [87]. The second linear regression model was a bivariate analysis of Number of COVID-19 Vaccines Received and Duration of COVID-19 Symptoms, based on the knowledge that COVID-19 disease severity may depend on whether one has received COVID-19 vaccinations, particularly the booster shot [67]. Again, additional independent variables were incrementally included or excluded in successive models.

## 3. Results

### 3.1. Demographics and Clinical Characteristics

Overall, 282 participants (age, median [range], 55 [18,19,20,21,22,23,24,25,26,27,28,29,30,31,32,33,34,35,36,37,38,39,40,41,42,43,44,45,46,47,48,49,50,51,52,53,54,55,56,57,58,59,60,61,62,63,64,65,66,67,68,69,70,71,72,73,74,75,76,77,78,79,80] years; 46.1% biologically male, 61.3% white) were considered in the complete case analysis. Supplementary Table S1 summarizes the demographics and clinical characteristics of the analyzed study population. Nearly half of the participants (46.8%) had never received any COVID-19 vaccinations by the end of the trial (May 2022); more than a quarter of the participants (26.6%) had never received OPV from childhood vaccinations nor from prior traveling; more than half of the participants (56.7%) reported exposures to the Omicron variant.

### 3.2. Outcome 1—Odds of Testing Positive for SARS-CoV-2 Infection (Aim 1)

Independent bivariate logistic regression models performed suggest that the odds of testing positive for SARS-CoV-2 infection may be influenced by SARS-CoV-2 variant strains, OPV vaccination, COVID-19 vaccination, and/or receipt of the COVID-19 booster vaccine (Supplementary Table S2).

Without adjusting for other variables, the odds of testing positive were 1.72 times higher for participants who had never received any COVID-19 vaccination (95%CI 1.05–2.83, *p* < 0.05) and 3.92 times higher for those who had never received OPV in their lifetime (95%CI 2.27–6.88, *p* < 0.001) compared with participants who had received such vaccinations. Moreover, those who received the COVID-19 booster vaccine showed a 55% reduction in odds of testing positive compared with participants who had never received any COVID-19 vaccinations (95%CI 0.23–0.84, *p* < 0.05). Additionally, the odds of testing positive were 1.79 times higher for those exposed to the Omicron variant compared with those exposed to the Delta variant (95%CI, 1.09–2.94, *p* < 0.05).

Whether one had received an influenza vaccination in the past year or a Tdap vaccination in the past 5 years did not significantly influence testing positive for SARS-CoV-2. Similarly, the duration between vaccinations (COVID-19, influenza, IPV on Day 1 of the clinical trial, or IPV/OPV prior to enrollment) and SARS-CoV-2 exposure did not significantly affect testing positive (Supplementary Table S2).

A phased approach of multivariate logistic regressions using forward selection revealed that the most parsimonious model of factors related to vaccination histories that influenced testing positive for SARS-CoV-2 included Exposure to SARS-CoV-2 Variant (Omicron/Delta), Number of COVID-19 Vaccines Received (0–3), and Previously Received OPV (Yes/No) (Table 2). All else equal, the odds of testing positive for SARS-CoV-2 were 3.92 times higher among participants who had never received OPV in their lifetime, either as a child or for travel purposes, compared with those who had previously received OPV (95%CI 2.22–7.03, *p* < 0.001). Participants who were exposed to the Omicron strain had 1.81 times higher odds of testing positive than those exposed to the Delta strain (95%CI 1.06–3.10, *p* < 0.05). Adjusting for SARS-CoV-2 variant strain exposure and OPV vaccination status, the number of COVID-19 shots (one, two, or three) did not significantly influence whether one tested positive for SARS-CoV-2 infection compared with participants who had never received COVID-19 vaccinations.

### 3.3. Outcome 2—Odds of Experiencing COVID-19 Symptoms (Aim 2)

Bivariate analyses of independent variables related to vaccination histories indicate that SARS-CoV-2 variant strain, OPV vaccination, COVID-19 vaccination, receipt of the COVID-19 booster vaccine, and the duration between vaccinations (COVID-19, influenza, and IPV) and SARS-CoV-2 exposure may influence whether COVID-19 symptoms are experienced (Supplementary Table S3).

Without adjusting for other factors, the odds of experiencing COVID-19 symptoms were 4.06 times higher among participants who had never received OPV (95%CI 2.35–7.17, *p* < 0.001) and 1.72 times higher among those who had never received any COVID-19 vaccination (95%CI 1.06–2.79, *p* < 0.05), compared to those who had received the vaccination. Participants who were fully vaccinated and boosted by the COVID-19 vaccine (three total shots) showed a 54% reduction in odds of experiencing COVID-19 symptoms compared with those who had never received any COVID-19 vaccinations (95%CI 0.25–0.84, *p* < 0.05). The specific SARS-CoV-2 strain to which participants were exposed, either Delta or Omicron, also influenced COVID-19 symptoms, with those exposed to Omicron showing a 62% reduction in odds of experiencing symptoms compared with those exposed to Delta (95%CI 0.23–0.61, *p* < 0.001).

Interestingly, bivariate analyses suggest that the odds of experiencing COVID-19 symptoms among participants who were exposed to SARS-CoV-2 between 3 and 6 months after COVID-19 vaccination and IPV vaccination were 2.41 and 2.33 higher, respectively, than participants who had been exposed between 6 and 12 months after vaccination (95%CI 1.08–6.02, *p* < 0.05; 95%CI 1.23–4.48, *p* < 0.05, respectively). Conversely, participants who were exposed to SARS-CoV-2 between 3 and 6 months after influenza vaccination showed a 57% reduction in odds of experiencing COVID-19 symptoms compared with those exposed between 6 and 12 months after vaccination (95%CI 0.18–0.98, *p* < 0.05). These associations did not remain statistically significant once the models were adjusted for SARS-CoV-2 variant strains.

To account for the influence of multiple factors, multivariate logistic regression models were conducted employing a phased approach with forward selection. The following independent variables were included in the final, most parsimonious model: Exposure to SARS-CoV-2 Variant (Omicron/Delta), Number of COVID-19 Vaccines Received (0–3), and Previously Received OPV (Yes/No) (Table 3). All else equal, the odds of experiencing COVID-19 symptoms were 4.45 times higher among participants who had never received OPV in their lifetime compared with those who had previously received OPV (95%CI 2.48–8.17, *p* < 0.001). Participants who were exposed to the Omicron strain showed a 65% reduction in odds of experiencing COVID-19 symptoms compared with those exposed to the Delta variant (95%CI 0.20–0.59, *p* < 0.001). Adjusting for SARS-CoV-2 variant strain exposure and OPV vaccination status, COVID-19 vaccination did not show a protective effect against experiencing COVID-19 symptoms. There were no significant differences in the odds of experiencing symptoms among participants who had never received COVID-19 vaccination compared to those who had received one, two, or even three COVID-19 shots.

### 3.4. Outcome 3—Duration of COVID-19 Symptoms (Average Number of Days, Aim 3)

None of the participants included in these analyses were hospitalized or died due to COVID-19; therefore, the participant-reported COVID-19 outcome related to disease severity is the number of days that any given participant experienced symptoms related to COVID-19.

Without accounting for other variables, bivariate analyses demonstrated that participants who had not received an influenza vaccination, oral poliovirus vaccination, or COVID-19 vaccination—specifically two and three COVID-19 shots—experienced COVID-19 symptoms for significantly longer than those who had received such vaccinations (3.27, 6.53, and 2.91 more days, respectively) (Supplementary Table S4). Participants exposed to the Omicron variant experienced COVID-19 symptoms for 2.56 fewer days than those exposed to the Delta variant (95%CI −4.88–−0.24, *p* < 0.05). Neither receipt of Tdap vaccination nor duration between individual vaccinations (i.e., COVID-19, influenza, or poliovirus) and SARS-CoV-2 exposures significantly influenced the duration of COVID-19 symptoms.

In the final, most parsimonious linear regression model chosen through a phased approach, three independent variables were included: Exposure to SARS-CoV-2 Variant (Omicron/Delta), Number of COVID-19 Vaccines Received (0–3), and Previously Received OPV (Yes/No) (Table 4). Those who were exposed to the Omicron strain experienced symptoms for 2.23 fewer days than those exposed to the Delta strain (95%CI −4.46–−0.1, *p* < 0.05). Most notably, participants who had never received OPV in their lifetime experienced COVID-19 symptoms for significantly longer (6.17 more days) than those who had received OPV as a child or for travel purposes (95%CI 3.68–8.67, *p* < 0.001). While other regression models analyzed the variable of time between prior poliovirus vaccination and SARS-CoV-2 exposure, for both the IPV injection received on Day 1 of the clinical trial and prior OPV or IPV inoculations from travel or childhood vaccinations prior to enrollment, neither variable significantly affected the impact of prior OPV vaccination on COVID-19 symptom duration. These results suggest that OPV may provide a protective effect, irrespective of the timing of prior vaccine administrations.

Finally, there was no significant difference in the number of days of COVID-19 symptoms among those who had never received COVID-19 vaccinations compared to those who were either fully vaccinated or boosted with COVID-19 vaccines (95%CI −5.60–0.06, *p* = 0.055; 95%CI −5.44–0.04, *p* = 0.053).

## 4. Discussion

### 4.1. Synthesis

This secondary data analysis demonstrates the importance of analyzing vaccination histories, since it reveals that prior vaccinations, such as OPV, can influence COVID-19 health outcomes.

Bivariate and multivariate logistic regressions showed that participants who had never received OPV in their lifetime had significantly higher odds of testing positive for SARS-CoV-2 infection, experiencing COVID-19 symptoms, and being symptomatic for a longer duration than those who had received OPV as part of their childhood vaccination shots and/or for travel. Adjusting for SARS-CoV-2 variant strains and COVID-19 vaccinations, participants who had never received OPV experienced COVID-19 symptoms for significantly longer than those who had received OPV. The number of years that had passed since prior poliovirus vaccinations (prior to enrollment) did not have a significant effect on COVID-19 outcomes, indicating that memory T-cells could be activated and/or that protective effects from OPV could possibly last a lifetime, at least among individuals who recently received IPV.

SARS-CoV-2 variant strain exposure also affected whether participants tested positive for SARS-CoV-2 (with higher odds if exposed to Omicron) or experienced COVID-19 symptoms (with higher odds of being symptomatic and for a longer duration if exposed to Delta). Adjusting for OPV vaccination status and SARS-CoV-2 variant strain exposure, COVID-19 vaccination and/or the number of COVID-19 shots (one, two, or three) did not significantly influence whether participants tested positive for SARS-CoV-2 infection or experienced COVID-19 symptoms compared with those who had never received any COVID-19 vaccinations. The association between COVID-19 vaccination and the outcome “Duration of COVID-19 Symptoms” was not statistically significant; however, participants who were fully vaccinated and/or boosted experienced symptoms for an average of 2.75 fewer days than those who had never received COVID-19 vaccinations.

The observed associations, namely between SARS-CoV-2 variant strains, OPV vaccination, and COVID-19 outcomes, corroborate findings from prior literature. The study’s initial hypothesis included that exposure to the Omicron variant may increase the odds of testing positive for SARS-CoV-2, as the strain has been noted as being more infectious [83,84]. Additionally, social behaviors, including large group gatherings and other in-person interactions, may have increased during the Omicron wave because risk mitigation measures were less stringent compared with the prior Delta period of the pandemic [88,89].

The findings also validate prior studies that suggest poliovirus vaccination may be associated with lower risks of SARS-CoV-2 infection and COVID-19 mortality [45,51,52,53,59]. While most studies have focused on OPV, participants enrolled in the clinical trial analyzed herein were inoculated with IPV. Since prior receipt of OPV significantly influenced participants’ COVID-19 outcomes, this begs the question: Are childhood OPV vaccinations enough to sustain protective effects throughout an individual’s lifetime, or does the immune system require a secondary challenge, either another dose of OPV or an introduction of IPV? If protective effects could have been achieved without a poliovirus vaccine booster, then it would have been expected that the majority of adults in the United States, those who would have received poliovirus vaccinations between 1963 and 1999, be more protected from SARS-CoV-2 than was the case. However, this study shows that OPV protective effects may be observed in individuals that have recently received IPV.

IPV alone does not induce as diverse an immunological response as OPV, which engages B-cells, T-cells, and innate immunity. Yet, studies have shown that IPV can significantly boost mucosal immunity and memory CD4+ T-cells among individuals who have previously received OPV, and thus are mucosally primed [90,91]. For instance, John et al., (2014) conducted a randomized open-label trial in which children who had previously received OPV were either given IPV or no vaccine. Both groups were subsequently given a second dose of OPV. Stool samples indicated that the IPV group shed significantly less virus than the group that did not receive a supplementary IPV dose [92]. Similarly, Jafari et al., (2014) found that one dose of IPV enhanced mucosal immunity in children primed with OPV compared with those who did not receive IPV. They also found that mucosal immunity wanes with age by observing that the 10-year-olds shed more virus than younger participants [93].

Altogether, the present analyses combined with evidence from prior studies reveal that IPV may be beneficial in boosting mucosal immunity and enabling protective effects against viral threats. Future trials should compare the effect of IPV inoculation in OPV-primed and OPV-naïve adults on COVID-19 outcomes. Since children receive four doses of poliovirus vaccinations, future studies should also explore how protective effects in adults may vary based on dosage and administration schedules. Participants in this study only received one dose of IPV, though preliminary in vitro data suggest that polio-immune serum samples from individuals who receive more than one dose may exhibit higher inhibition of SARS-CoV-2 cytopathic effects by disrupting RdRp activities and viral replication processes [59]. Subsequent studies should examine not only clinical outcomes but also antibody levels and mechanistic processes on a cellular level.

While the initial hypothesis projected that receipt of vaccinations, including influenza, COVID-19, and Tdap, as well as durations between vaccinations and SARS-CoV-2 exposures may impact the odds of testing positive, experiencing symptoms, or the duration of COVID-19 symptoms, the multivariate regression models did not reveal such influences. There were no significant differences between participants who had recently received influenza, COVID-19, or Tdap vaccinations and those who had not, nor between those who had received vaccinations greater or less than 6 months prior to SARS-CoV-2 exposures, when antibodies are known to wane [66,70,71,74,76,77]. Future trials should investigate whether IPV inoculation may complement other vaccines, such as influenza and COVID-19, and boost antibodies for a longer duration.

### 4.2. Strengths and Limitations

While the initial study design serves as a strong foundation, there are opportunities to strengthen the research further in subsequent trials. While most studies examining IPV introduction to mucosally-primed individuals focus on children who have received OPV, this study was the first one to apply the concept to adults. While the study population was small and limited to one geographic region, the single-arm study design in which all participants received IPV was designed with intention as a pilot, proof-of-concept study to assess poliovirus vaccination in the context of the COVID-19 pandemic. However, future, larger studies should go further by comparing COVID-19 outcomes between adults who have received IPV and those who have not. Such studies should also include analyses of serological data to further validate participant-reported outcomes and/or identify those who may be positive for SARS-CoV-2 infection but who are asymptomatic. Individuals with asymptomatic infection were only identified in this study if they happened to test themselves due to knowledge of a possible exposure.

Although understanding COVID-19 outcomes depended on participant response to the 6- and 12-month follow-up phone calls, the attrition rate was merely 6%, indicating that both attrition bias and the impact of missing data was likely negligible. Survivor bias was also deemed highly improbable, as only two out of the 300 participants (0.67%) passed away during the course of the study due to causes unrelated to COVID-19. Additionally, the potential for recall bias was minimized, as participants were instructed to contact the clinical trial staff if they had been exposed to SARS-CoV-2 and/or tested positive, enabling COVID-19 status reports to be more accurate and verified during the 6- and 12-month phone calls. Vaccination History reports were also confirmed by the clinical site staff using vaccination registry records and databases, as well as copies of vaccination cards provided by the participants, if available. Some childhood vaccinations could not be verified by state records in the case where vaccines had been administered more than 60 years prior to the study, but those participants had vivid recollections of receiving sugar cubes at school, a distinctive memory from typical arm injections. More recent inoculations were more easily accessible through online records and registries.

Another factor to consider is that individuals who receive vaccinations may be more health conscious or engage in different socio-behavioral health practices than the norm. While this influence may apply to COVID-19 or influenza vaccinations, it is not applicable to childhood poliovirus vaccinations, as every state has a poliovirus vaccine mandate for childcare and elementary school requirements [94]. The type of poliovirus vaccination administered, IPV or OPV, would depend on national guidelines at the time, and thus would not be affected by individual health behaviors.

### 4.3. Conclusions and Implications for Research and Practice

Current COVID-19 vaccines target the surface spike protein, which evolves over time and may render vaccines that were developed for a prior strain less successful. To induce antibodies that may continue to be effective regardless of strain, new therapeutics should target highly conserved, non-surface proteins, such as RdRp. While drugs that target RdRp, such as remdesivir and molnupiravir, are being used to treat patients who have COVID-19, prophylactic measures such as vaccines that target this core protein have yet to be developed. However, structural similarities between RNA viruses suggest potential for cross-reactivity and repurposing of existing vaccines that induce antibodies against shared highly conserved proteins.

This secondary data analysis examined vaccination histories among clinical trial participants who received IPV and found that OPV-naïve participants had significantly higher odds of testing positive for SARS-CoV-2 infection, experiencing COVID-19 symptoms, and experiencing symptoms for a longer duration. These findings highlight the importance of considering not only vaccination histories but also the concept of mucosal priming. As seen in prior studies, introduction of IPV can boost mucosal immunity in individuals who have previously received OPV. Mucosal immunity is critical in protecting individuals against viruses that infect mucosal surfaces, such as poliovirus and SARS-CoV-2; therefore, a boost in such immunity could have vast implications.

In addition to complementing existing COVID-19 vaccines, the introduction of IPV among OPV-primed individuals could provide an extra layer of protection in economically disadvantaged settings and countries. As inequitable vaccine distribution persists across the globe, and some geographic regions ration their resources and available vaccines to prioritize high-risk populations (i.e., healthcare workers, immunocompromised individuals, individuals who have not acquired natural COVID-19 infection), access to an existing vaccine would provide opportunities to protect populations in a more equitable manner [95].

Since most economically disadvantaged countries utilize OPV for their childhood vaccination schedules, it is likely that most individuals will already be mucosally primed and, thus, only require an IPV booster [96]. For countries where citizens are not mucosally primed, OPV and IPV inoculations may be required to achieve comparable levels of immunity, but the majority of countries that solely utilize IPV are industrialized nations with access to COVID-19 vaccines and other resources [97,98]. If future studies validate the data presented here, public health agencies may encourage policies to expand the usage of poliovirus vaccines, and existing systems and resources could be used to roll out mass vaccination campaigns and access. While production would have to be ramped up in facilities that currently manufacture OPV and/or IPV for childhood vaccinations, factors that posed logistical difficulties for COVID-19 vaccine administration, such as those related to transportation, storage, and training, would not necessarily apply to this vaccine rollout [99]. For instance, poliovirus vaccines can be transported in refrigerated units, which not only means a decrease in costs compared with other vaccines requiring specialty freezers but also promotes an expansion in geographic access, as vaccines can be transported to farther and/or remote areas [100]. Additionally, the poliovirus vaccine can be stored for 2 years and will not expire within hours, minimizing waste and logistical concerns [101]. Finally, because the poliovirus vaccine is already widely used for children, less training would be required for healthcare professionals, who not only have experience administering the vaccine but also understand the history and adverse effects associated with it.

Looking beyond the potential use in low-resource areas, poliovirus vaccination may also be beneficial in countries that have sufficient resources. Individuals living in communities that are inequitably affected by upstream health factors, such as structural and systemic barriers to preventive medical care access, may be less likely to receive vaccines that are not mandatory. People with vaccine hesitancy may be more comfortable with a well-established vaccine that they already received as children (poliovirus vaccine) than a new vaccine that was rapidly developed (COVID-19 vaccine). Disparities in vaccination uptake must be considered, and a subsequent analysis of the data will focus on social determinants of health, including age, education, health insurance, and other socio-demographic or health-related variables.

Future, larger trials should further explore how poliovirus vaccinations may mitigate the risk of SARS-CoV-2 infection and COVID-19 symptoms, if the effects vary based on IPV or OPV administration, and whether a dose-response relationship exists with different dosing schedules. If such studies confirm the findings and trends presented herein, public health agencies should consider implementing a poliovirus booster vaccination rollout, with IPV for those who are mucosally primed and OPV/IPV for OPV-naïve individuals. By utilizing a vaccine that induces antibodies against the highly conserved RdRp protein, a more robust immunological response may be achieved in combination with currently available COVID-19 vaccines, which do not induce antibodies against the SARS-CoV-2 N-protein nor any other nonsurface proteins [102]. Of course, a COVID-19 specific vaccine that not only targets core proteins but also provides mucosal immunity would be ideal to prevent infection and mitigate disease severity against emerging variants. However, until such a vaccine is developed, there is an opportunity to repurpose an existing vaccine that could boost mucosal immunity in vulnerable populations.

## Figures and Tables

**Table 1:** Independent variables.

Variable	Variable Type	Levels
Years Between Last Poliovirus Vaccination (IPV or OPV) Prior to Enrollment and SARS-CoV-2 Exposure	Continuous	N/A (continuous, 13–68)
Months Between Last COVID-19 Vaccination and SARS-CoV-2 Exposure	Categorical	3 levels (0–3 months; 3–6 months; 6–12 months)
Months Between Last Influenza Vaccination and SARS-CoV-2 Exposure	Categorical	3 levels (0–3 months; 3–6 months; 6–12 months)
Months Between Last IPV Poliovirus Vaccination (Day 1) and SARS-CoV-2 Exposure	Categorical	3 levels (0–3 months; 3–6 months; 6–12 months)
Number of COVID-19 Vaccines Received	Ordinal	4 levels (None, Not Vaccinated; One, Partially Vaccinated; Two, Fully Vaccinated; Three, Fully Vaccinated + Boosted)
Received COVID-19 Vaccine in the Last Year	Dichotomous	2 (Yes; No)
Received Influenza Vaccine in the Last Year	Dichotomous	2 (Yes; No)
Received Tetanus-Diphtheria-Pertussis (Tdap) Vaccine in the Last 5 Years	Dichotomous	2 (Yes; No)
Previously Received Oral Poliovirus (OPV) Vaccine	Dichotomous	2 (Yes; No)
Exposed to Omicron or Delta Strain	Dichotomous	2 (Yes; No)

**Table 2:** Final logistic regression model for ‘Tested Positive for SARS-CoV-2’ outcome.

Independent Variables	Adjusted Odds Ratio	95% Confidence Interval	*p*-Value
(ref)	1.0	---	---
Exposed to Omicron Strain	1.81	1.06–3.10	<0.05
Number of COVID-19 Vaccines Received *			
One (Partially Vaccinated)	2.07	0.66–6.84	0.214
Two (Fully Vaccinated)	0.64	0.32–1.26	0.207
Three (Fully Vaccinated + Boosted)	0.60	0.30–1.16	0.136
Previously Received Oral Poliovirus (OPV) Vaccine (No)	3.92	2.22–7.03	<0.001

* Reference group: Individuals who had not been administered COVID-19 vaccinations (N = 132).

**Table 3:** Final logistic regression model for ‘Experienced COVID-19 Symptoms’ outcome.

Independent Variables	Adjusted Odds Ratio	95% Confidence Interval	*p*-Value
(ref)	1.0	---	---
Exposed to Omicron Strain	0.35	0.20–0.59	<0.001
Number of COVID-19 Vaccines Received *			
One (Partially Vaccinated)	2.33	0.73–8.30	0.164
Two (Fully Vaccinated)	0.64	0.32–1.26	0.203
Three (Fully Vaccinated + Boosted)	0.66	0.34–1.27	0.214
Previously Received Oral Poliovirus (OPV) Vaccine (No)	4.45	2.48–8.17	<0.001

* Reference group: Individuals who had not been administered COVID-19 vaccinations (N = 132).

**Table 4:** Final linear regression model for ‘Duration of COVID-19 Symptoms’ outcome.

Independent Variables	ß Coefficient	95% Confidence Interval	*p*-Value
Exposed to Omicron Strain	−2.23	−4.46–−0.1	<0.05
Number of COVID-19 Vaccines Received			
One (Partially Vaccinated)	2.37	−2.77–7.51	0.365
Two (Fully Vaccinated)	−2.77	−5.60–0.06	0.055
Three (Fully Vaccinated + Boosted)	−2.72	−5.44–0.04	0.053
Previously Received Oral Poliovirus (OPV) Vaccine (No)	6.17	3.68–8.67	<0.001

## Data Availability

The datasets analyzed for this study may be available upon request.

## Supplementary Materials

The following supporting information can be downloaded at: https://www.mdpi.com/article/10.3390/vaccines12030219/s1, Table S1: Participant demographics and characteristics, Table S2: Main effects of predictor variables on Testing Positive for SARS-CoV-2, Table S3: Main effects of predictor variables on Experiencing COVID-19 Symptoms, Table S4: Main effects of predictor variables on the Number of Days COVID-19 Symptoms Were Experienced.
