# Supplementary material for: Inactivated Poliovirus Vaccine Booster Reduces the Likelihood of COVID-19 Outcomes in Individuals Primed with Oral Poliovirus Vaccination
Source: Vaccines (Basel). 2024 Feb 20;12(3):219. doi: 10.3390/vaccines12030219 (PMC10974902; doi:10.3390/vaccines12030219)
Supplement: Supplementary file 1 [file vaccines-12-00219-s001.zip › vaccines-2803364-supplementary.pdf]

vaccines

MDPI

Supplementary Materials

Supplementary Figures and Tables

Table S1. Participant demographics and characteristics

Characteristic	Frequency (%) (N = 282)	Characteristic	Frequency (%) (N = 282)
Age (years)		Months Between Last COVID-19 Vaccination and SARS-CoV-2 Exposure	
Mean	51.4	0-3 Months	61 (21.6)
Median	55.0	3-6 Months	35 (12.4)
Range	18-80	6-12 Months	54 (19.1)
Biological Sex		Months Between Last Influenza Vaccination and SARS-CoV-2 Exposure	
Male	130 (46.1)	0-3 Months	56 (19.9)
Female	152 (53.9)	3-6 Months	62 (22.0)
Race/Ethnicity		6-12 Months	38 (13.5)
White	173 (61.3)	Months Between Last IPV Polio Vaccination (Day 1) and SARS-CoV-2 Exposure	
Hispanic/Latinx	67 (23.8)	0-3 Months	7 (2.5)
Asian	26 (9.2)	3-6 Months	46 (16.3)
Other	16 (5.7)	6-12 Months	229 (81.2)
Received COVID-19 Vaccine In the Last Year, n (%)	150 (53.2)	Years Between Last Polio Vaccination (IPV or OPV) prior to enrollment and SARS-CoV-2 Exposure	
Pfizer-BioNTech	69 (24.5)	Median	47.0
Moderna	71 (25.2)	25% Quartile	30.3
Janssen (Johnson & Johnson)	10 (3.5)	75% Quartile	57.0
Number of COVID-19 Vaccines Received		Exposed to Omicron or Delta SARS-CoV-2 Strain	
None (Not Vaccinated)	132 (46.8)	Omicron	160 (56.7)
One (Partially Vaccinated)	14 (4.9)	Delta	122 (43.3)
Two (Fully Vaccinated)	62 (22.0)	Tested Positive for SARS-CoV-2	
Three (Fully Vaccinated + Boosted)	74 (26.2)	Yes	99 (35.1)
Previously Received Oral Polio Vaccine (OPV)		No	183 (64.9)
Yes	207 (73.4)	Experienced COVID-19 Symptoms	
No	75 (26.6)	Yes	111 (39.4)
Received Influenza Vaccine In the Last Year		No	171 (60.6)
Yes	156 (55.3)	Duration of COVID-19 Symptoms	
No	126 (44.7)	Mean	10.3 days
Received DTAP Vaccine In the Last Five Years		Median	7.0 days
Yes	131 (46.5)	Range	1 day - 120 days $^{(N=1)}$
No	151 3.5)		

Table S2. Main effects of predictor variables on Testing Positive for SARS-CoV-2

Independent Variables	Unadjusted Odds Ratio	95% Confidence Interval	P-value	Independent Variables	Unadjusted Odds Ratio	95% Confidence Interval	P-value
Received COVID-19 Vaccine In the Last Year				Months Between Last COVID-19 Vaccination and SARS-CoV-2 Exposure			
Yes	1.0	---	---	6-12 Months	1.0	---	---
No	1.72	1.05 – 2.83	<0.05	0-3 Months	1.47	0.63 – 3.48	0.376
Received Influenza Vaccine In the Last Year				3-6 Months	2.33	0.92 – 6.02	0.075
Yes	1.0	---	---	Months Between Last Influenza Vaccination and SARS-CoV-2 Exposure			
No	1.27	0.77 – 2.07	0.345	6-12 Months	1.0	---	---
Received DTAP Vaccine In the Last Five Years				0-3 Months	0.81	0.34 – 1.94	0.637
Yes	1.0	---	---	3-6 Months	0.76	0.32 – 1.79	0.523
No	0.94	0.57 – 1.53	0.800	Months Between Last IPV Poliovirus Vaccination (Day 1) and SARS-CoV-2 Exposure			
Previously Received Oral Poliovirus (OPV) Vaccine				6-12 Months	1.0	---	---
Yes	1.0	---	---	0-3 Months	0.32	0.02 – 1.93	0.299
No	3.92	2.27 – 6.88	<0.001	3-6 Months	1.49	0.78 – 2.83	0.225
Number of COVID-19 Vaccines Received				Years Between Last Poliovirus Vaccination (IPV or OPV) prior to enrollment and SARS-CoV-2 Exposure			
None (Not Vaccinated)	1.0	---	---	Years	1.00	0.98 – 1.01	0.833
One (Partially Vaccinated)	1.87	0.62 – 5.96	0.272	Exposed to Omicron or Delta Strain			
Two (Fully Vaccinated)	0.57	0.29 – 1.08	0.092	Delta	1.0	---	---
Three (Fully Vaccinated + Boosted)	0.45	0.23 – 0.84	<0.05	Omicron	1.79	1.09 – 2.94	<0.05

Table S3. Main effects of predictor variables on Experiencing COVID-19 Symptoms

Independent Variables	Unadjusted Odds Ratio	95% Confidence Interval	P-value	Independent Variables	Unadjusted Odds Ratio	95% Confidence Interval	P-value
Received COVID-19 Vaccine In the Last Year				Months Between Last COVID-19 Vaccination and SARS-CoV-2 Exposure			
Yes	1.0	---	---	6-12 Months	1.0	---	---
No	1.72	1.06 – 2.79	<0.05	0-3 Months	1.39	0.62 – 3.18	0.422
Received Influenza Vaccine In the Last Year				3-6 Months	2.41	1.08 – 6.02	<0.05
Yes	1.0	---	---	Months Between Last Influenza Vaccination and SARS-CoV-2 Exposure			
No	1.16	0.71 – 1.87	0.556	6-12 Months	1.0	---	---
Received DTAP Vaccine In the Last Five Years				0-3 Months	0.46	0.20 – 1.07	0.073
Yes	1.0	---	---	3-6 Months	0.43	0.18 – 0.98	<0.05
No	0.92	0.57 – 1.48	0.726	Months Between Last IPV Poliovirus Vaccination (Day 1) and SARS-CoV-2 Exposure			
Previously Received Oral Poliovirus (OPV) Vaccine				6-12 Months	1.0	---	---
Yes	1.0	---	---	0-3 Months	1.34	0.26 – 6.24	0.703
No	4.06	2.35 – 7.17	<0.001	3-6 Months	2.33	1.23 – 4.48	<0.05
Number of COVID-19 Vaccines Received				Years Between Last Poliovirus Vaccination (IPV or OPV) prior to enrollment and SARS-CoV-2 Exposure			

None (Not Vaccinated)	1.0	---	---	Years	1.00	0.98 – 1.01	0.584
One (Partially Vaccinated)	2.10	0.69 – 7.13	0.206	Exposed to Omicron or Delta Strain			
Two (Fully Vaccinated)	0.55	0.29 – 1.03	0.068	Delta	1.0	---	---
Three (Fully Vaccinated + Boosted)	0.46	0.25 – 0.84	<0.05	Omicron	0.38	0.23 – 0.61	<0.001

Table S4. Main effects of predictor variables on the Number of Days COVID-19 Symptoms Were Experienced

Independent Variables	ß Coefficient	95% Confidence Interval	P-value	Independent Variables	ß Coefficient	95% Confidence Interval	P-value
Received COVID-19 Vaccine In the Last Year				Months Between Last COVID-19 Vaccination and SARS-CoV-2 Exposure			
No	3.27	0.99 – 5.56	<0.01	0-3 Months	1.43	-2.15 – 5.02	0.432
Received Influenza Vaccine In the Last Year				3-6 Months	2.20	-1.97 – 6.37	0.299
No	2.91	0.61 – 5.21	<0.05	Months Between Last Influenza Vaccination and SARS-CoV-2 Exposure			
Received DTAP Vaccine In the Last Five Years				0-3 Months	-1.21	-5.26 – 2.84	0.556
No	0.63	-1.69 – 2.95	0.596	3-6 Months	-1.87	-5.84 – 2.10	0.354
Previously Received Oral Poliovirus (OPV) Vaccine				Months Between Last IPV Poliovirus Vaccination (Day 1) and SARS-CoV-2 Exposure			
No	6.53	4.03 – 9.04	<0.001	0-3 Months	-0.42	-7.84 – 7.01	0.912
Number of COVID-19 Vaccines Received				3-6 Months	2.98	-0.15 – 6.11	0.062
One (Partially Vaccinated)	2.10	-3.26 – 7.46	0.441	Years Between Last Poliovirus Vaccination (IPV or OPV) prior to enrollment and SARS-CoV-2 Exposure			
Two (Fully Vaccinated)	-3.46	-6.39 – -0.52	<0.05	Years	0.02	-0.06 – 0.10	0.636
Three (Fully Vaccinated + Boosted)	-4.14	-6.91 – -1.37	<0.01	Exposed to Omicron or Delta Strain			
				Omicron	-2.56	-4.88 – -0.24	<0.05

vaccines
